# Dietary Ursolic Acid Supplementation Induces Beneficial Changes in Skeletal Muscle mRNA Expression and Improves Functional Mobility in Older Cats with Sarcopenia: A Proof-of-Concept Study

**DOI:** 10.3390/ani16213377

**Published:** 2026-10-26

**Authors:** Scott M. Ebert, Celine S. Nicolas, Andrew R. Judge, Jaime G. Lopez, Alan T. Taylor, Kotha Sekharam, John J. Talley, Christophe A. Rème, Christopher M. Adams

**Affiliations:** 1 Emmyon, Inc., Rochester, MN 55902, USA; scott-ebert@emmyon.com (S.M.E.); andrew-judge@emmyon.com (A.R.J.); john-talley@emmyon.com (J.J.T.); 2 Division of Endocrinology, Diabetes, Metabolism, and Nutrition, Department of Medicine, and Department of Biochemistry and Molecular Biology, Mayo Clinic, Rochester, MN 55902, USA; 3 Global Marketing and Market Development, Virbac SA, 06511 Carros, France; christophe.reme@virbac.com; 4 Department of Physical Therapy, Myology Institute, and Cancer Institute, University of Florida, Gainesville, FL 32610, USA; 5 US Petcare Innovation, Virbac NA, Westlake, TX 76262, USA; jaime.lopez@virbacus.com (J.G.L.); alan.taylor@virbacus.com (A.T.T.); 6 Florida Research Group, LLC, Tampa, FL 33610, USA; kotha@frgvet.com

**Keywords:** Ursolic acid, Ursolyx, feline, muscle health, muscle atrophy, sarcopenia, cachexia, mobility, mobility improvement, exercise, dietary supplement

## Abstract

Cats frequently lose mobility when they become older, ill, or injured, leading to additional impairments in health and quality of life. To help address this problem, a dietary supplement for cats containing ursolic acid was tested. Ursolic acid is a natural dietary compound that promotes muscle health and mobility in several non-feline species. A 60-day proof-of-concept efficacy study of ursolic acid dietary supplementation was conducted in older domestic shorthair cats with sarcopenia (age-related skeletal muscle atrophy). In feline skeletal muscle, 12 mg of ursolic acid per day generated beneficial changes in skeletal muscle mRNA expression, including repression of mRNAs that promote muscle atrophy and weakness and induction of mRNAs that promote muscle health. Furthermore, ursolic acid generated significant improvements in several important measures of feline mobility, including jumping, playing with toys, rising from a resting position, and transitioning from a standing position to a sitting or lying position. These findings identify ursolic acid supplementation as a new nutritional approach for promoting beneficial molecular and functional mobility outcomes in cats.

## Simple Summary

Loss of mobility is a highly prevalent, unsolved problem in feline care. To help address this problem, the effects of ursolic acid were tested in cats. Ursolic acid is a natural dietary compound that promotes muscle health and mobility in several non-feline species. In older cats with age-related muscle atrophy (sarcopenia), 60 days of ursolic acid supplementation at a dose of 12 mg per day was well tolerated and generated beneficial changes in skeletal muscle mRNA expression and significant improvements in mobility. These data identify a new nutritional approach for promoting and maintaining muscle health and mobility in cats.

## 1. Introduction

As cats become older, ill, or injured, they frequently experience a profound deterioration in skeletal muscle structure and function, leading to significant impairments in mobility, activity, and quality of life [1,2,3,4,5,6]. Some of the most common causes of muscle dysfunction and mobility loss in cats include advanced age, cancer, chronic kidney disease, hyperthyroidism, diabetes, malnutrition, and muscle disuse related to osteoarthritis, trauma, or obesity [7]. Unfortunately, the loss of muscle health and mobility in cats is highly prevalent, especially as cats live longer due to advances in other areas of feline care. Furthermore, this decline is often multifactorial in nature, with illness or injury superimposed on a background of advanced age, and it is frequently irreversible due to the absence of muscle-directed pharmacologic therapies or reliable non-pharmacologic approaches [7].

Identifying ways to protect and promote muscle health and strength in companion animals is an area of growing interest. Skeletal muscle atrophy involves and requires induction of mRNAs that promote muscle atrophy and weakness (pro-atrophy mRNAs) and repression of mRNAs that are essential for the normal structure and function of skeletal muscle (anti-atrophy mRNAs) [8,9,10,11,12].

Ursolic acid is a pentacyclic triterpene acid that is naturally found in a wide range of edible fruits and herbs, including apples, cranberries, blueberries, plums, pears, rosemary, sage, holy basil, thyme, and oregano [13]. Ursolic acid was originally discovered as a natural dietary compound for muscle health through laboratory-based studies in mouse models and cultured skeletal myotubes [14,15,16,17,18]. These studies demonstrated that ursolic acid acts directly on skeletal muscle, where it promotes anabolic signaling and blunts catabolic signaling, leading to increased strength, specific force, endurance exercise capacity, and muscle mass, as well as protection against muscle atrophy and weakness during advanced age, fasting, muscle disuse, and cancer [14,15,16,17,18].

In skeletal muscle of dogs and mice, ursolic acid inhibits atrophy-associated changes in mRNA expression, leading to repression of pro-atrophy mRNAs and de-repression of anti-atrophy mRNAs [14,17,18,19]. In older dogs with age-related skeletal muscle atrophy (also known as sarcopenia), ursolic acid supplementation was well tolerated, favorably modified mRNA expression, and improved voluntary exercise participation and mobility, supporting its use as a safe dietary compound to promote musculoskeletal function [19].

In the current study, we tested the hypothesis that ursolic acid might generate beneficial changes in skeletal muscle mRNA expression in cats and improvements in mobility in a 60-day proof-of-concept efficacy study in older cats with sarcopenia.

## 2. Materials and Methods

### 2.1. Ursolic Acid Dietary Supplement

The ursolic acid dietary supplement (URSOLYX™ Soft Chews for Cats) was provided by Virbac (Westlake, TX, USA) and created in collaboration with Emmyon, Inc. (Rochester, MN, USA), with manufacturing and co-development performed by Florida Research Group (Tampa, FL, USA). Each soft chew weighed 1 g and contained 12 mg of ursolic acid from a natural herbal source, 18% crude protein, 0.3% crude fiber, 21% crude fat, and excipients formulated to enhance oral bioavailability and palatability in cats. The amount of ursolic acid (12 mg) was derived via body surface area allometric scaling from previous efficacy studies in dogs, mice, and humans [14,15,17,18,19,20,21,22,23].

### 2.2. Study Design

To evaluate the efficacy of ursolic acid supplementation in cats, we conducted a 60-day proof-of-concept study in older domestic shorthair cats with mild to moderate sarcopenia (age-related skeletal muscle atrophy). The study was designed to test the hypothesis that ursolic acid supplementation in cats generates beneficial changes in skeletal muscle mRNA expression and improves mobility. Since skeletal muscle mRNA expression and mobility can vary widely between individuals, the study was designed to rely upon paired intrasubject longitudinal controls, allowing us to compare final values to initial baseline values in each animal.

The study also evaluated tolerability and safety of ursolic acid supplementation over the course of this 60-day study. Tolerability and safety were assessed through daily health observations, formal clinical examinations, and clinical chemistry and hematology assessments.

The study enrolled 20 female and male shorthair domestic cats (*Felis catus*). All cats had mild to moderate sarcopenia (age-related skeletal muscle atrophy) based on the WSAVA muscle condition score (https://wsava.org/wp-content/uploads/2020/01/Muscle-Condition-Score-Chart-for-Cats.pdf, accessed on 22 October 2026), consistent with their age. They were deemed to be otherwise healthy by a veterinarian based on clinical examination, body condition score (between 3 and 4 on a 5-point scale for all cats), laboratory testing, and exclusion of concurrent disease. All cats were spayed or neutered.

The study facility (Clinvet, Bloemfontein, South Africa) complied with the latest version of South African National Standard (SANS) 10386 (“The care and use of animals for scientific purposes”). The study protocol was approved by the Virbac North America Ethical Review Committee and by the study facility’s Institutional Animal Care and Use Committee (IACUC), whose members had authority to inspect the study site and animals at will. All procedures were designed to avoid or minimize discomfort, distress, and pain to the animals. To maintain social welfare standards mandated by the IACUC, cats were group-housed in social units with ad libitum access to water and commercial cat food. Group housing precluded quantification of individual daily food and protein intake, though total room food consumption was monitored daily.

Cats were divided into two age-matched and weight-matched groups, with 10 cats per group. Cats meeting all health inclusion criteria were assigned to groups based on pre-study screening, identifying animals that voluntarily accepted the soft chew treat, ensuring stress-free delivery of the ursolic acid supplement. Because the study relied on paired intra-subject longitudinal controls, baseline sex distribution was not used as a blocking variable during allocation. The control group (8.6 ± 1.0 years old; 3.7 ± 0.8 kg; 4M/6F) received no supplement. The treatment group (7.6 ± 1.4 years old; 4.1 ± 0.5 kg; 8M/2F) received one soft chew (12 mg of ursolic acid) daily for 60 days.

All study personnel (veterinarians, laboratory analysts, and mobility assessors) were strictly blinded to group allocation, except for the technicians administering the treats. Thus, while chew administration was open-label, all clinical evaluators, mobility scoring technicians, and laboratory analysts remained strictly blinded to group allocation throughout the study. Health observations were performed daily, and formal clinical examinations were conducted on study days −21, −7, 14, 35, and 58. Blood samples were collected on days −7 and 59; muscle biopsies were collected on days −1 and 60; and mobility assessments were performed on days −6 to −2 and 54 to 58.

### 2.3. Blood Collection and Analyses

Blood specimens (6 mL per cat) were collected via jugular venipuncture on study days −7 and 59. Blood was collected prior to any sedation or surgical interventions in order to prevent anesthetic interference with laboratory parameters. Samples were analyzed for clinical chemistry (glucose, electrolytes, urea nitrogen, creatinine, total protein, albumin, AST, ALT, and alkaline phosphatase) on a Beckman Coulter AU480 analyzer (Beckman Coulter, Inc., Brea, CA, USA) and complete blood counts with differentials on a Sysmex XN-1000V analyzer (Sysmex Corporation, Kobe, Japan).

### 2.4. Skeletal Muscle Biopsies and Analysis of Skeletal Muscle mRNA Expression

To test the hypothesis that ursolic acid might have beneficial effects in feline skeletal muscle, quadriceps muscle biopsies were obtained from each cat at the beginning and end of the study. RNA was then isolated from the skeletal muscle biopsies, and levels of atrophy-associated mRNAs were quantified. Intrasubject changes in mRNA levels in each cat over the course of the 60-day study were then analyzed.

Quadriceps muscle biopsies were obtained on study days −1 and 60 under sedation with intramuscular medetomidine and ketamine. The dorsolateral cranial thigh was shaved and surgically prepared. On day −1, a baseline biopsy was taken from the quadriceps muscle (*m. vastus lateralis*). On day 60, a repeat biopsy was obtained from the contralateral limb to eliminate confounding transcriptional signatures associated with local tissue healing. A 6 mm biopsy punch was used to collect ~10 mg of tissue, which was flash-frozen and stored at −80 °C. Sample yield was restricted to ~10 mg to minimize surgical trauma per institutional welfare guidelines, which precluded parallel tissue histology or mitochondrial assays. Sedation was reversed with intramuscular atipamezole, and cats received postoperative analgesia (subcutaneous meloxicam daily for 2 days) and systemic antibiotics (intramuscular penicillin G daily for 3 days).

RNA extraction, library preparation, sequencing, and bioinformatic analysis were conducted by Azenta Life Sciences (South Plainfield, NJ, USA). Total RNA was extracted using Qiagen RNeasy Plus Universal mini kits (Qiagen, Hilden, Germany). RNA quantity and integrity were confirmed via Qubit 2.0 (Life Technologies, Carlsbad, CA, USA) and Agilent TapeStation 4200 (Agilent Technologies, Palo Alto, CA, USA). Poly(A)-selected libraries were prepared using NEBNext Ultra II RNA Library Prep Kits (NEB, Ipswich, MA, USA) and sequenced on an Illumina NovaSeq X+ instrument (San Diego, CA, USA) (2 × 150 bp paired-end). Reads were trimmed with Trimmomatic v0.36 and mapped to the *F. catus* reference genome (Fca126_mat1.0) using the STAR aligner v2.5.2b. Exonic unique gene hit counts were calculated via featureCounts (Subread v1.5.2). Normalized counts and log2 fold changes were generated using DESeq2 via Wald tests. Sample-level sequencing depth, read quality metrics, trimming yield, and STAR reference genome mapping rates are detailed in Supplementary Table S1.

### 2.5. Mobility Assessments

Mobility assessments were performed by placing each cat for 30 min in a cage fitted with resting benches of different heights and toys for the cat to play with and for the assessor to entice the cat. In each session, cats were assessed for their ability to jump to any height, their ability to jump up at least 1 m, their ability to jump down at least 1 m from the ground, their ability to groom, their ability to play with toys, their ability to rise from a resting position, and their ability to lie or sit down. These mobility assessments were chosen because they capture a range of voluntary movements that are typical of cats.

In each assay, mobility was graded on an ordinal scale: 4 (normal), 3 (impaired), 2 (more impaired), 1 (almost absent), and 0 (absent). Scoring was performed by three technicians who were blinded to group allocation throughout the trial. Baseline mobility assessments were performed on study days −6 to −2 and averaged to obtain the baseline value. Mobility assessments were then repeated on study days 54 to 58 and averaged to obtain the final value.

### 2.6. Statistics

Statistical analyses were performed with GraphPad Prism version 11.1.0 using statistical tests described in the table legend and figure legends. *p*-values less than 0.05 were considered significant.

## 3. Results

### 3.1. Tolerability and Safety Monitoring of Ursolic Acid Supplementation in Older Cats with Sarcopenia

Ursolic acid supplementation was well tolerated and generated no adverse effects in the cats that were enrolled in this 60-day study. Daily observations and formal veterinary examinations on study days 14, 35, and 58 revealed no concerning clinical findings. Body weight slightly increased in both groups by day 58, with no significant difference between groups (mean increase of 0.14 kg and 0.30 kg in the control and test groups respectively). Venous blood samples were collected from each cat at the beginning and end of the study, and glucose and electrolyte levels, renal and hepatic function markers, and complete blood counts remained within normal reference ranges (Table 1).

### 3.2. In Older Cats with Sarcopenia, Dietary Supplementation with Ursolic Acid Generates Beneficial Changes in Skeletal Muscle mRNA Expression

**Figure 1:**
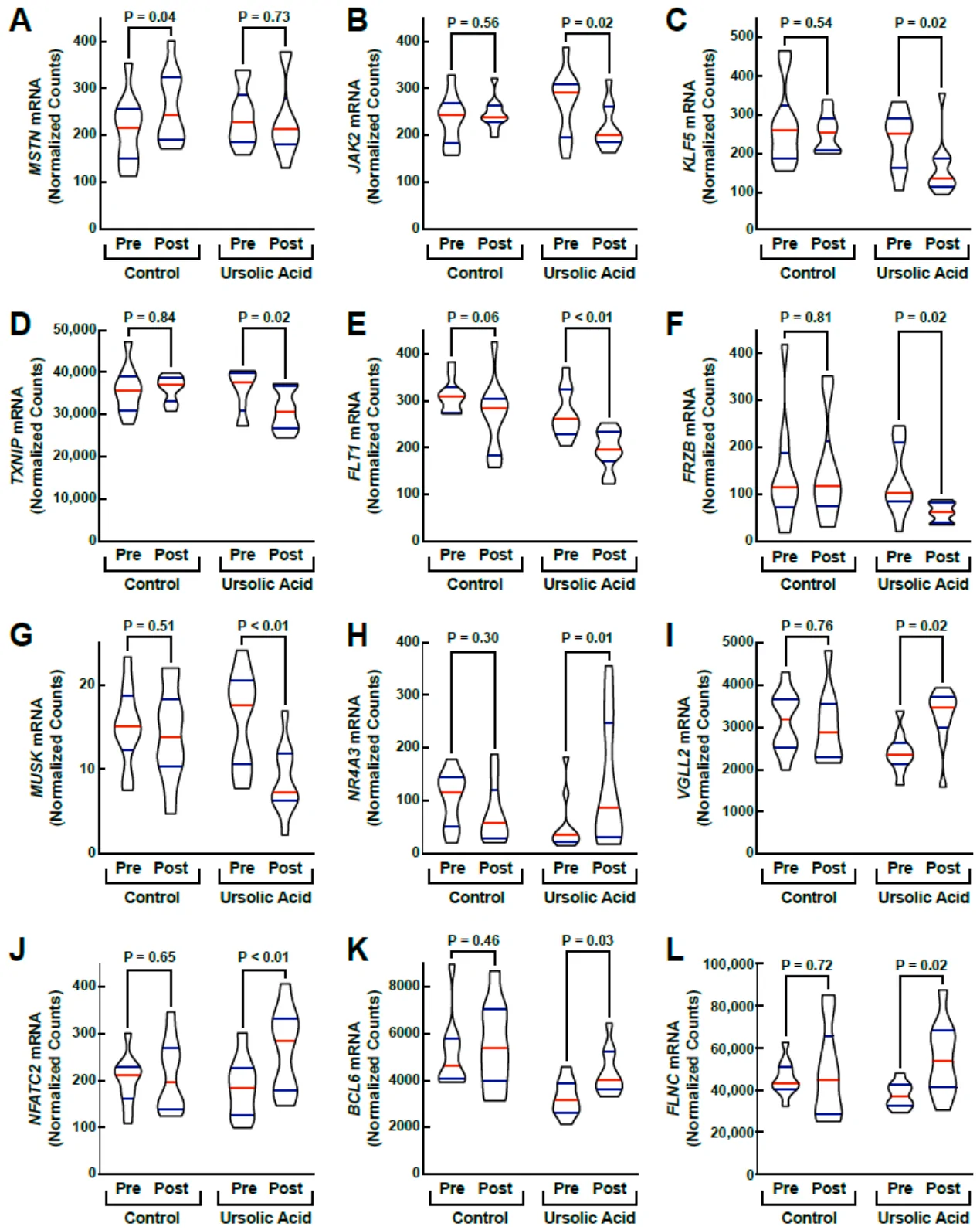
In older cats with sarcopenia, dietary supplementation with ursolic acid generates beneficial changes in skeletal muscle mRNA expression. Domestic cats with mild to moderate sarcopenia received either no dietary supplement or one ursolic acid soft chew (12 mg of ursolic acid) once a day for 60 days. Quadriceps muscle biopsies were obtained from each animal on study day −1 (“Pre”) and on study day 60 (“Post”). RNA was isolated from the muscle biopsies and used for quantification of mRNAs encoding mediators and markers of muscle atrophy and weakness (including *MSTN* (**A**), *JAK2* (**B**), *KLF5* (**C**), *TXNIP* (**D**), *FLT1* (**E**), *FRZB* (**F**), *MUSK* (**G**)) and mRNAs encoding proteins that protect against muscle atrophy and weakness (including *NR4A3* (**H**), *VGLL2* (**I**), *NFATC2* (**J**), *BCL6* (**K**), and *FLNC* (**L**)). Data are truncated violin plots from 10 control cats and 10 ursolic acid-supplemented cats, with red bars denoting median values, blue bars denoting interquartile ranges, and *p*-values determined by two-way ANOVA with post-hoc multiple comparison corrections using the two-stage linear step-up procedure of Benjamini, Krieger, and Yekutieli. *MSTN*: Myostatin; *JAK2*: Janus Kinase 2; *KLF5*: KLF Transcription Factor 5; *TXNIP*: Thioredoxin Interacting Protein; *FLT1*: FMS-Related Receptor Tyrosine Kinase 1; *FRZB*: Frizzled Related Protein; *MUSK*: Muscle Associated Receptor Tyrosine Kinase; *NR4A3*: Nuclear Receptor Subfamily 4 Group A Member 3; *VGLL2*: Vestigial Like Family Member 2; *NFATC2*: Nuclear Factor of Activated T Cells 2; *BCL6*: BCL6 Transcription Repressor; *FLNC*: Filamin C.

Ursolic acid generated changes in feline skeletal muscle mRNA expression that are known to promote muscle health and protect against muscle atrophy and weakness. For example, in feline skeletal muscle, ursolic acid prevented induction of *MSTN* mRNA, which encodes myostatin, a secreted protein that decreases muscle mass and strength [24,25] (Figure 1A). Ursolic acid also repressed other mRNAs that encode mediators or biomarkers of muscle atrophy and weakness, including but not limited to *JAK2* mRNA (which encodes a tyrosine kinase that promotes muscle atrophy and weakness [26,27]), *KLF5* mRNA (which encodes a zinc-finger transcription factor that promotes muscle atrophy and weakness [28]), *TXNIP* mRNA (which encodes an oxidative stress sensor that promotes muscle atrophy and weakness [29]), *FLT1* mRNA (which encodes a protein that reduces skeletal muscle vascularization and blood flow [30,31]), and *FRZB* and *MUSK* mRNAs (markers of skeletal muscle denervation and denervation-induced muscle atrophy and weakness [32,33]) (Figure 1B–G). 

In conjunction with its repression of pro-atrophy mRNAs, ursolic acid significantly increased anti-atrophy mRNAs that are essential for the normal structure and function of skeletal muscle. These included but were not limited to *NR4A3* mRNA (which encodes NOR1, a nuclear hormone receptor that promotes mitochondrial function and insulin sensitivity and protects against muscle atrophy and weakness [34,35,36]), *VGLL2* mRNA (which encodes a skeletal muscle-specific transcriptional cofactor that protects against muscle atrophy and weakness [37,38,39]), *NFATC2* mRNA (which encodes a calcium-responsive transcription factor that protects against muscle atrophy and weakness [40]), *BCL6* mRNA (which encodes a transcriptional repressor that protects against muscle atrophy and weakness [41,42]), and *FLNC* mRNA (which encodes a critical structural protein that protects against muscle atrophy and weakness [43,44,45]) (Figure 1H–L).

**Figure 2:**
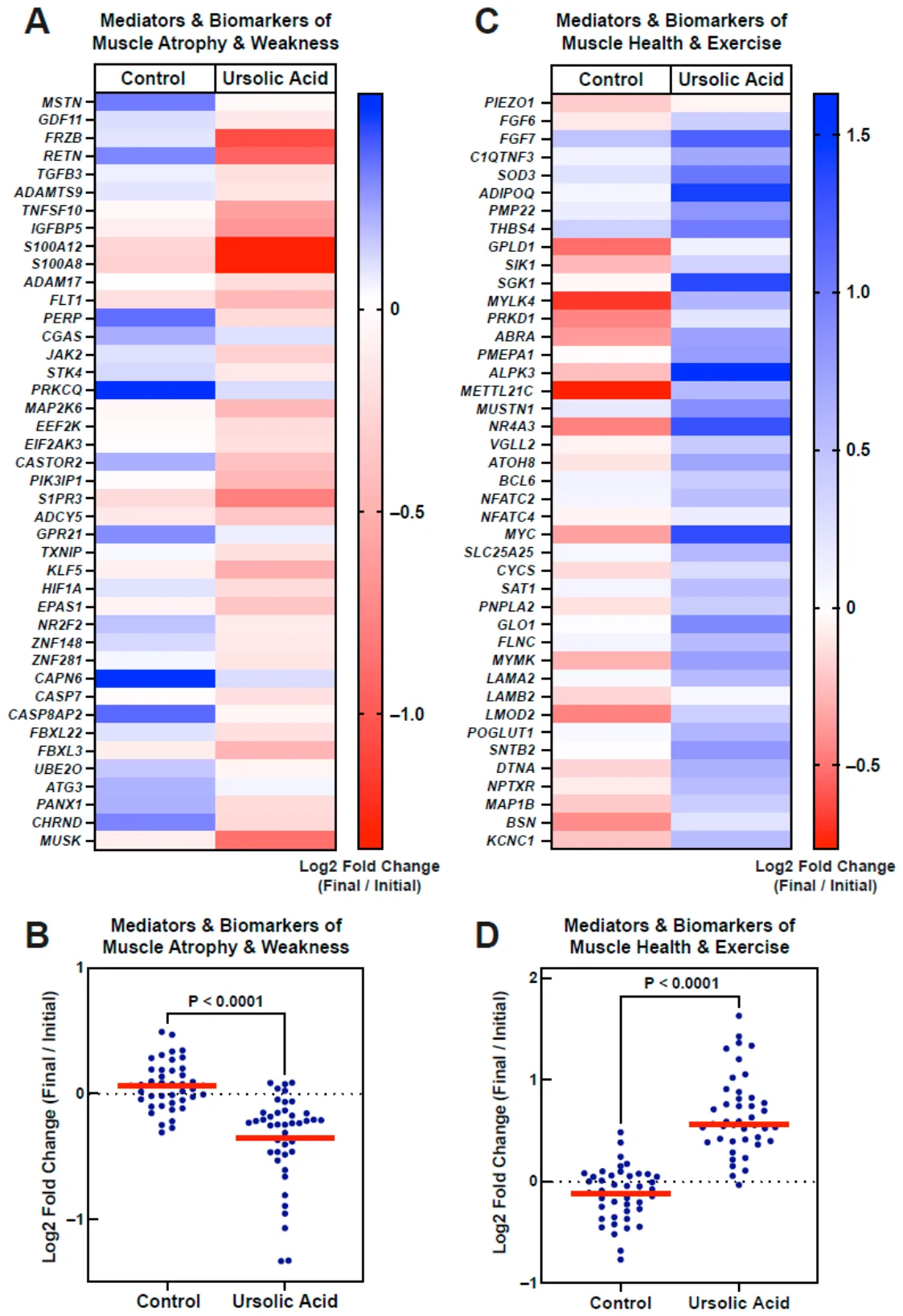
In feline skeletal muscle, ursolic acid decreases mRNAs encoding mediators and biomarkers of atrophy and weakness and increases mRNAs encoding mediators and biomarkers of muscle health and exercise. Domestic cats with mild to moderate sarcopenia received either no dietary supplement or one ursolic acid soft chew (12 mg of ursolic acid) once a day for 60 days. Quadriceps muscle biopsies were obtained from each animal on study day −1 (“Initial”) and on study day 60 (“Final”). RNA was isolated from the muscle biopsies, and log2 fold changes in the indicated mRNA levels were determined in each animal by comparing final mRNA levels to initial baseline mRNA levels in the same animal. Data are from 10 control cats and 10 ursolic acid-supplemented cats. (**A**,**B**) Mean log2 fold changes in mRNAs encoding mediators and biomarkers of muscle atrophy and weakness, shown as a heat map (**A**) and scatter plot (**B**). (**C**,**D**) Mean log2 fold changes in mRNAs encoding mediators and biomarkers of muscle health and exercise, shown as a heat map (**C**) and scatter plot (**D**). In (**B**,**D**), each data point represents the mean log2 fold change in one mRNA; horizontal lines denote means of all assessed mRNA levels, and two-tailed *p*-values were determined with paired *t*-tests.

Altogether, ursolic acid significantly decreased or tended to decrease levels of over 40 mRNAs that encode mediators or biomarkers of muscle atrophy and weakness (Figure 2A,B), while simultaneously increasing or tending to increase over 40 mRNAs that encode mediators or biomarkers of muscle health and exercise (Figure 2C,D).

### 3.3. Dietary Supplementation with Ursolic Acid Improves Mobility in Older Cats with Sarcopenia

**Figure 3:**
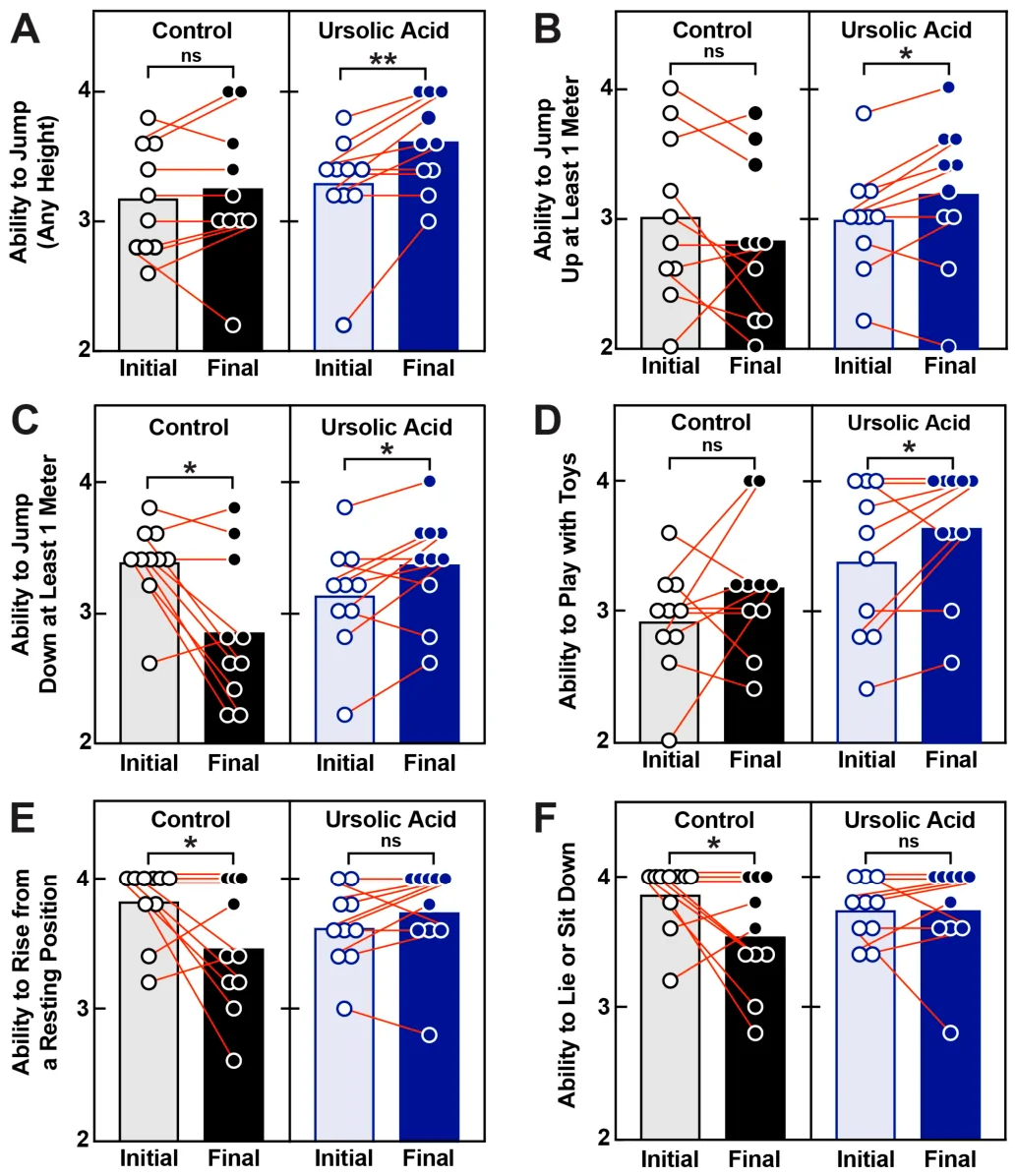
Ursolic acid improves mobility in cats. Domestic cats with mild to moderate sarcopenia received either no dietary supplement or one ursolic acid soft chew (12 mg of ursolic acid) once a day for 60 days. In each cat, mobility assessments were performed on study days −6 to −2 and averaged to obtain the baseline value (“Initial”), then repeated on study days 54 to 58 and averaged to obtain the final value (“Final”). In each assay, mobility was graded on the following scale: 4 (normal), 3 (impaired), 2 (more impaired), 1 (almost absent), and 0 (absent). (**A**) Ability to jump to any height. (**B**) Ability to jump up at least 1 m. (**C**) Ability to jump down at least 1 m. (**D**) Ability to play with toys. (**E**) Ability to rise from a resting position. (**F**) Ability to lie or sit down. (**A**–**F**) Each data point represents data from one cat, red lines denote paired values from the same cat, and bars indicate mean values. One-tailed *p*-values were determined with Wilcoxon matched-pairs signed-rank tests. * *p* < 0.05; ** *p* < 0.01; ns: *p* > 0.05.

In four of the seven mobility tests (ability to jump to any height, ability to jump up at least 1 m, ability to jump down at least 1 m, and ability to play with toys), ursolic acid significantly improved performance over the course of the 60-day study, whereas performance in the control group declined or did not change (Figure 3A–D). In two mobility tests (ability to rise from a resting position and ability to lie or sit down), performance in the control group significantly declined over the course of the 60-day study, and ursolic acid prevented the decline (Figure 3E,F). In the seventh test (ability to groom), performance significantly improved in both groups (not shown). These results indicate that ursolic acid significantly improves mobility in older cats, consistent with the molecular effects of ursolic acid in feline skeletal muscle.

## 4. Discussion

The current study identifies ursolic acid as a natural dietary compound that can help improve functional mobility in aging cats. We investigated the effects of ursolic acid in older cats with sarcopenia and found that it generates several beneficial molecular changes in feline skeletal muscle, including repression of over 40 mRNAs that encode mediators and biomarkers of muscle atrophy and weakness and de-repression of over 40 mRNAs that encode mediators and biomarkers of muscle health and exercise. Most importantly, the molecular effects of ursolic acid in feline skeletal muscle are associated with significant improvements in mobility, including jumping, playing, and moving between standing and recumbent positions. These results identify ursolic acid supplementation as a new nutritional approach for promoting beneficial molecular and functional mobility outcomes in cats.

This study also showed that ursolic acid supplementation was well tolerated by the cats involved in this study. These data are consistent with formal safety data collected in a separate internal study of 24 cats that were administered either 0 (control), 12 mg (recommended dose) or 60 mg (five-fold dose) of ursolic acid formulated in the same soft chew as the one used in this study for 28 days, which also showed no adverse effects in these cats.

The overall pattern of ursolic acid-induced molecular changes in feline skeletal muscle suggests a shift from a catabolic, inflamed, hypoxia-stressed, growth-restrained state (characteristic of normal muscle aging) to a more oxidative, insulin-sensitive, fatigue-resistant and structurally reinforced state with improved innervation, metabolic health, strength, and muscle quality. These findings are consistent with previous studies of ursolic acid in other species, which demonstrated that ursolic acid inhibits multiple catabolic signaling pathways in skeletal muscle (including the myostatin, FOXO, ATF4-C/EBPβ, STAT3, and NF-kB pathways) while simultaneously promoting anabolic signaling pathways in skeletal muscle (including insulin and IGF-1 pathways), ultimately leading to increased expression of anabolic/anti-atrophy mRNAs and decreased expression of catabolic/pro-atrophy mRNAs [14,15,17,18,19,22,23]. The current data from feline skeletal muscle, coupled with previous data from non-feline species, suggest a model in which ursolic acid promotes a broad range of beneficial molecular changes that help optimize skeletal muscle structure and function and counteract effects of aging.

The broad molecular effects of ursolic acid are also consistent with the way it was originally identified as a natural dietary compound for muscle health [14]. Ursolic acid was identified by searching for small molecules whose global effects on mRNA expression in human cell lines negatively correlate with the total transcriptional output of all catabolic/pro-atrophy signaling pathways that are induced and all anabolic/anti-atrophy signaling pathways that are repressed in human and mouse skeletal muscle under two diverse atrophy conditions (prolonged fasting and spinal cord injury) [14]. That unbiased, systems-based screen revealed ursolic acid as a small molecule that could potentially counteract the complex mixture of positive and negative transcriptional changes that are central to the pathogenesis of skeletal muscle atrophy and weakness [14], which was subsequently confirmed through experimental studies in multiple mouse models of muscle atrophy [14,15,17,18,22,23], older dogs with sarcopenia [19], and now older cats with sarcopenia.

Strengths of this study include the integration of blinded functional mobility scoring with paired intra-subject transcriptomic profiling. In addition, the study employed intrasubject controls and sensitive molecular assays that are well-suited for a small proof-of-concept study in subjects with diverse characteristics. Furthermore, the finding that ursolic acid improves mobility in aging cats represents a meaningful real-world, quality-of-life outcome for cats and pet owners and is therefore an important advance for feline health.

The current study also has several limitations. First, while functional mobility represents a clinically relevant endpoint in feline geriatrics, this initial proof-of-concept study lacked quantitative imaging of skeletal muscle mass. Second, muscle biopsy yield was deliberately restricted to ~10 mg to minimize animal discomfort, and although that yield was sufficient for assessments of mRNA expression, it was too small to permit assessments of skeletal muscle histology, contractility, or mitochondrial function. Third, social group-housing prevented individual quantification of food and protein intake. Fourth, baseline sex distribution was unbalanced between groups. Fifth, inter-observer variability for mobility scoring was not formally calculated. Based on these considerations, it will be important to follow up this initial proof-of-concept study with future studies that utilize larger, sex-stratified cohorts of older cats, randomized, double-blind, placebo-controlled designs, and direct assessments of feline skeletal muscle mass, skeletal muscle fiber size, skeletal muscle contractility, skeletal muscle mitochondrial function, and skeletal muscle protein expression.

Additional studies of ursolic acid are also now warranted in other conditions that impair muscle health and mobility in cats, including cancer, chronic kidney disease, hyperthyroidism, diabetes, malnutrition, and muscle disuse related to osteoarthritis, trauma, or obesity. In mouse models of several of these disorders, including cancer [18,23], chronic kidney disease [22], malnutrition [14], muscle disuse [14], and obesity [15], ursolic acid significantly improves skeletal muscle mass and function.

## 5. Conclusions

The current results of this proof-of-concept study indicate that dietary supplementation with ursolic acid could improve skeletal muscle health and mobility in older cats with sarcopenia. While these findings need to be confirmed in larger-scale randomized controlled trials, they identify a new nutritional approach for promoting and maintaining muscle health and mobility in cats.

## Figures and Tables

**Table 1:** Effects of ursolic acid on glucose and electrolyte levels, renal and hepatic function, and complete blood counts. Domestic cats with mild to moderate sarcopenia received either no dietary supplement or one ursolic acid soft chew (12 mg of ursolic acid) once a day for 60 days. Venous blood samples were collected from each animal on study day −7 (“Initial”) and study day 59 (“Final”) and subjected to quantitative assessments of glucose and electrolyte levels, renal and hepatic function, and complete blood counts. Data are means ± SD from 10 animals per condition. Shapiro–Wilk tests were used to assess the distribution of the data. Two-tailed *p*-values were determined with Student’s *t*-tests. * *p* < 0.05 vs. initial value. All values in all cats remained within the reference ranges. None of the small changes that were statistically significant based on *p*-values were considered to be clinically relevant by veterinarians who were caring for the cats and performing close clinical examinations throughout the study. AST: aspartate aminotransferase; ALT: alanine aminotransferase; Alk Phos: alkaline phosphatase; RBCs: red blood cells; MCV: mean corpuscular volume; MCH: mean corpuscular hemoglobin; MCHC: mean corpuscular hemoglobin concentration; WBCs: white blood cells.

Parameter	Control (No Chew)		Ursolic Acid (12 mg/Day)		Reference Range
	Initial	Final	Initial	Final	
Glucose (mmol/L)	4.6 ± 0.5	4.6 ± 0.9	5.0 ± 1.5	4.4 ± 0.7	4.0–9.7
Sodium (mmol/L)	152 ± 1	150 ± 3 *	152 ± 1	151 ± 2	147–157
Potassium (mmol/L)	4.9 ± 0.6	4.6 ± 0.2	4.6 ± 0.3	4.6 ± 0.2	3.7–5.2
Chloride (mmol/L)	119 ± 2	118 ± 2	120 ± 3	118 ± 2	114–126
Calcium (mmol/L)	2.3 ± 0.1	2.3 ± 0.1	2.2 ± 0.1	2.3 ± 0.1 *	2.0–2.8
Magnesium (mmol/L)	0.82 ± 0.05	0.85 ± 0.07	0.81 ± 0.07	0.85 ± 0.07 *	0.74–1.00
Phosphorus (mmol/L)	1.6 ± 0.2	1.5 ± 0.2	1.4 ± 0.2	1.5 ± 0.2	0.93–2.00
Urea (mmol/L)	8.2 ± 1.4	8.6 ± 1.1	7.5 ± 0.9	7.5 ± 0.8	5.7–13.2
Creatinine (µmol/L)	92.7 ± 26.8	106.7 ± 25.6 *	79.7 ± 20.9	104.6 ± 12.9 *	80.0–203.0
Total Protein (g/L)	70.5 ± 5.3	73.8 ± 5.9 *	74.0 ± 3.5	78.0 ± 4.4 *	63.0–88.0
Albumin (g/L)	28.8 ± 1.9	27.2 ± 1.7 *	27.1 ± 2.5	27.3 ± 3.1	26.0–39.0
AST (U/L)	25.2 ± 7.2	27.4 ± 9.1	23.4 ± 4.0	23.6 ± 4.0	16.0–67.0
ALT (U/L)	52.4 ± 15.9	52.7 ± 17.8	45.4 ± 16.1	45.6 ± 17.2	27.0–158.0
Alk Phos (U/L)	35.1 ± 19.4	38.0 ± 22.0	39.7 ± 14.7	39.3 ± 13.9	12.0–59.0
Hemoglobin (g/dL)	13.3 ± 1.3	14.0 ± 0.9	13.2 ± 1.6	14.0 ± 1.6	10.3–16.2
Hematocrit (%)	39.7 ± 4.2	42.8 ± 3.4 *	39.4 ± 4.6	42.7 ± 4.8 *	28.2–52.7
RBCs (10^12^/L)	8.9 ± 1.0	9.2 ± 0.8	8.7 ± 1.3	9.2 ± 1.3	7.1–11.5
MCV (fL)	44.8 ± 2.0	46.5 ± 2.6 *	45.6 ± 3.3	46.5 ± 3.0	39.0–56.0
MCH (pg)	15.0 ± 0.8	15.2 ± 0.9	15.3 ± 1.1	15.2 ± 1.1	13.0–17.0
MCHC (g/dL)	33.5 ± 1.3	32.7± 1.2 *	33.6 ± 0.8	32.7 ± 0.8 *	28.5–37.8
Reticulocytes (10^9^/L)	17.4 ± 11.0	17.3 ± 8.1	23.4 ± 12.6	18.0 ± 5.4	9.0–61.0
Platelets (10^9^/L)	338 ± 123	219 ± 53 *	267 ± 108	225 ± 107 *	155–641
WBCs (10^9^/L)	10.2 ± 3.9	11.2 ± 3.0	11.3 ± 3.2	10.8 ± 2.2	3.9–19.0
Neutrophils (10^9^/L)	5.9 ± 2.9	6.4 ± 2.3	6.9 ± 2.8	5.9 ± 1.5	2.62–15.2
Lymphocytes (10^9^/L)	3.3 ± 0.9	3.6 ± 1.1	3.2 ± 1.0	3.8 ± 1.1 *	0.85–5.85
Monocytes (10^9^/L)	0.29 ± 0.14	0.28 ± 0.13	0.51 ± 0.37	0.43 ± 0.26	0.04–0.53
Eosinophils (10^9^/L)	0.67 ± 0.32	0.88 ± 0.70	0.62 ± 0.25	0.69 ± 0.39	0.09–2.18
Basophils (10^9^/L)	0.02 ± 0.03	0.02 ± 0.01	0.03 ± 0.03	0.03 ± 0.02	0.00–0.10

## Data Availability

The data presented are available on request from celine.nicolas@virbac.com.

## Supplementary Materials

The following supporting information can be downloaded at: https://www.mdpi.com/article/10.3390/ani16213377/s1. Table S1: RNA-Seq Quality Control.
