# Supplementary material for: Dietary Ursolic Acid Supplementation Induces Beneficial Changes in Skeletal Muscle mRNA Expression and Improves Functional Mobility in Older Cats with Sarcopenia: A Proof-of-Concept Study
Source: Animals (Basel). 2026 Oct 26;16(21):3377. doi: 10.3390/ani16213377 (PMC13761402; doi:10.3390/ani16213377)
Supplement: Supplementary file 1 [file animals-16-03377-s001.zip › animals-4610144-supplementary.pdf]

Dietary ursolic acid supplementation induces beneficial changes in skeletal muscle mRNA expression and improves functional mobility in older cats with sarcopenia: A proof-of-concept study

Supplemental data

Table S1: RNA-Seq Quality Control

	Average	SD
**Total Reads (M)**	39834050	5548522
**Total Mapped Reads (M)**	37323030	5209642
**% Total Mapped Reads**	93.69	0.76
**Unique Mapped Reads**	33462886	4733688
**% Unique Mapped Reads**	83.99	1.05
**Mean Quality Score (Q-Score)**	38.53	0.10
**% Bases > Q30**	92.90	0.44
